# The “Dutch Reading Test for Adults” has Been Used for 29 Years to Estimate the Premorbid Performance Level, does it Still Meet the Expectations?

**DOI:** 10.5334/pb.1136

**Published:** 2022-08-22

**Authors:** Noortje Hermans, Jean-Philippe van Dijck

**Affiliations:** 1 Thomas More University College of Applied Sciences, department of Applied Psychology, Antwerp, Belgium; 2 Ghent University, department of Experimental Psychology, Ghent, Belgium

**Keywords:** premorbid functioning, hold task, Nederlandse Leestest voor Volwassenen, NLV, NART, premorbid IQ

## Abstract

To detect cognitive change after brain damage, it is important to know the level of premorbid intellectual functioning. A popular instrument in this context is the “Nederlandse Leestest voor Volwassenen” (NLV; Schmand et al., 1992). In this test, 50 words with irregular pronunciation must be read out loud. The score on this test used to be considered as a good estimate of someone’s premorbid IQ, due to high correlations with the Verbal and Full-Scale IQ estimates of the WAIS (Verbal IQ: r = .85, Full Scale IQ: r = .74; Schmand et al., 1992). Despite some updates of the normative data (e.g., Bouma et al., 2012), the validity of the test has not been re-evaluated since. The aim of the current study was to evaluate whether the NLV still correlates sufficiently with the WAIS-IV to warrant its proper use as a psychodiagnostic tool. In Study 1, 30 participants (age range: 20–29 year) were tested, and in Study 2, 51 participants (age range: 45–65 year). We checked whether the NLV-generated IQ-score estimates correlated with the different IQ indices of the WAIS-IV. In the younger group, no correlations were found between the NLV-generated IQ-score estimate and any of the WAIS-IV indices nor the Full-Scale IQ. In the older group, the NLV-generated IQ-score estimate correlated with Full scale IQ and the indices of Verbal Comprehension, Working Memory and Processing Speed. These correlations were all <.46 which is far below the typically hold standard in test development of >.70. Based on these findings we conclude the NLV in its current form is not appropriate anymore to estimate premorbid IQ in both young and older adults.

## Introduction

After brain damage (from mild to severe), many patients report cognitive problems. This deterioration is often persistent and interferes with daily life activities and professional functioning. For this reason, neurologists often refer these patients to a clinical neuropsychologist for a profound assessment of their cognitive functioning to make a prognosis, to determine the need for cognitive rehabilitation (and its content) and/ or to use this information for health insurance related issues (Lambrecht & Hermans, 2019).

During a neuropsychological assessment, a battery of tests is typically administered which covers several cognitive domains (like memory, attention, executive functioning). To facilitate interpretation, the different test scores are considered in the light of norms that take gender, age, and education level of the patient into account. While it is important to determine where the test scores are situated within the respected norm group, it does not necessarily give a good indication about the degree of cognitive decline. After all, it is possible that the brain lesioned patient achieves within the average range compared to the used normative sample, but that this level of functioning reflects a significantly reduced performance compared to its premorbid functioning. It could also be the other way around. Neuropsychological testing could reveal performance within the lower range, which is actually in accordance with the patient’s original level of functioning. It is thus only when mapping the difference between the premorbid and the actual level of functioning, that we can obtain an indication of the cognitive decline in a particular patient (Green et al., 2008). Unfortunately, in clinical practice, results from assessments that took place before the injury are rarely available (Lambrecht & Hermans, 2019). For that reason, in most cases premorbid functioning must be estimated.

Various methods exist to estimate a person’s premorbid level of functioning (see Lezak et al., 2012 for an overview). A particular type of test in this context is the hold-test. A hold-test estimates premorbid intelligence based on cognitive tasks which strongly correlate with measurements of general intelligence, and which have proven to be resistant to aging and (mild) brain damage (Bouma et al., 2012; Hendriks et al., 2014; Kessels et al., 2012). These tests mainly measure stored knowledge and skills, such as vocabulary knowledge and reading skills. It has been shown that the performance on vocabulary tests is highly correlated with educational attainment, which in turn is a good predictor of premorbid functioning (Lezak et al., 2012; McGurn et al., 2004). Reading tests, in which irregularly spelled words must be pronounced correctly, are also highly correlated with verbal intelligence (Kessels et al., 2012; Schmand et al., 1991). This would be because more intelligent people often have a higher level of education and are therefore more familiar with irregularly spelled words (Smith-Seemiller et al., 1997). The mechanism behind word reading is believed to rely on two routes: a lexical (semantic) route and a direct non-lexical route. To correctly read irregularly spelled words, the lexical route must be activated, as the word needs to be recognized and the correct pronunciation be retrieved from memory (which drives on highly automated processes). If someone cannot rely on this lexical route when reading the irregularly spelled words (because this kind of words are not known to the person), the direct route must be used. Reading via this route relies on the grapheme-phoneme rule system, which is common to a language, but which does not contain information about irregularly spelled words. As such reading such words via this route which will lead to an incorrect pronunciation (Coltheart et al., 2001). Importantly, our ability to correctly pronounce known irregularly spelled words remains intact for a very long time with normal aging (Schmand et al., 1998) and with various (degenerative) brain disorders and damage (e.g. McGurn et al., 2004). As a result, the ability to read irregularly spelled words is typically considered as a good tool to estimate premorbid (verbal) intelligence (Bouma et al., 2012).

A popular reading test in this context is “The National Adult Reading Test” (NART, Nelson, 1982) which is frequently used in both clinical and research settings. This test consists of a list of 50 irregularly pronounced words (organized from easy to difficult) which must be read out loud by the patient, who gets points for each correctly pronounced word. Later the sum of these points is transformed to a score, which is as an estimate of someone’s Verbal and Full-Scale IQ. Importantly, this test has good psychometric properties, as in healthy subjects the estimated IQ scores obtained with this reading test correlate highly with scores obtained with regular intelligence tests. For example, studies with the (English version of the) NART found correlations between the performance on this task and the Full-Scale IQ obtained with the WAIS and WAIS-R in the range of r = .71 to .81 (Lezak et al., 2012). Correlations with Verbal IQ seem slightly higher, while those with Performal IQ are typically lower (e.g. Blair & Spreen, 1989; r = .83, .40, and .75, for Verbal, Performal and Full-Scale IQ respectively). Recently, the NART is restandardized against the WAIS-IV by Bright et al. (2018) who again found a strong correlation between the NART and the WAIS-IV Full Scale IQ (r = .69) and moderate correlations with the other WAIS-IV index scores (between .35 < r > .65) in a group of 92 neurologically healthy participants. They concluded that the NART is still an important tool for estimating premorbid functioning.

Given its usefulness, the test is translated to different languages and is used throughout the world (Lezak et al., 2012). The task is also translated to Dutch and is frequently used in the Netherlands and in the Dutch speaking part of Belgium (i.e., Flanders). The Dutch version goes under the name “the Dutch Reading Test for Adults” or “de Nederlandse Leestest voor Volwassenen” (abbreviated NLV; Schmand et al., 1992). The test is published in the Netherlands (where also the norms were collected), where the test used to have good psychometric properties (i.e., sufficient levels of reliability and validity). Schmand et al. (1992) tested 20 neurologically healthy participants and found significant correlations between performance on the NLV and the WAIS (respectively r = .85, .51 .and r = .74 for Verbal IQ, Performal IQ and Full-Scale IQ subscales). Updated norms were constructed later, taking age and gender into account (Bouma et al., 2012; Mulder et al., 1996) and additional scorings instructions were made for the Flemish population (Ceuppens, 2014 Appendix B).

Despite its frequent use in clinical and scientific contexts in both the Netherlands and Flanders, the validation studies and the collection of norms date back from 20 to 30 years ago. This is problematic, as it is known that the psychometric qualities of psychodiagnostic tests can deteriorate over time (Evers et al., 2010; Flynn, 1987). Furthermore, given the age of studies, the test has never been standardized against the more recent version of the WAIS (here the WAIS-IV; Wechsler, 2012). It is therefore necessary to re-investigate its validity (see e.g., Flynn, 2009). This is particularly important for reading tests because it is known that language and vocabulary knowledge are subject to changes over time due to, e.g., globalization, education, or the digitalization of our society (Crystal, 2012).

The aim of the present study is to investigate whether the current version of the Dutch reading test for adults (NLV) still has a good validity when standardized against the WAIS-IV. A test can be considered a valid measurement of premorbid functioning if: (1) the test results are related to intellectual ability as measured by intelligence tests, (2) the test gives a good estimate of the intelligence level in healthy people, and (3) the test is not sensitive to the effects of neurological and psychiatric disorders (Bouma, 2012). In the current study we re-investigate the first two criteria. As language knowledge is dynamic over time (Crystal, 2012), two studies were conducted in which the participants differed at least one generation in age (15 years). The first group consisted of young adults (age range 20–29 years) and the second group consisted of adults ranging from 45–65 years. From the recent validation study of the NART (Bright et al., 2018), it can be predicted that the task still correlates with the Full-Scale IQ and the Verbal Comprehension Index of the WAIS-IV. Additionally, we predict that the correlation will be more pronounced in the older age group compared to the young adults. Many words of the NLV are borrow-words from English, a language where young adults are more familiar with (making it possible that some of the test-items might have lost their discriminative power). In a first step, these correlations will be calculated on the norm scores obtained from both tasks. Second, as we collected data in Flanders (while the normative data were collected in the Netherlands), this correlation will be recalculated using the raw data of both tasks. This should allow us to evaluate the validity of the task, independently from the existing norms.

## Study 1: The Validity of the NLV in 20–29 Year Old’s

### Participants

Thirty subjects participated in this study (15 females, 15 males). All participants had Belgian nationality and Dutch as native language. Their average age was 23.633 year (SD = 2.414; range 20–29 years). This sample was heterogeneous in terms of intelligence, as the average WAIS-IV Full-Scale IQ was 102.133 (SD = 12.153; range between 79 and 123; see Table 1). The subjects self-declared that they had no history of neurological or psychological problems and had normal or corrected-to-normal vision. They all participated voluntarily without financial compensation and filled in an informed consent before participation. The participants were recruited and tested by two students in applied psychology of Thomas More and contributed to the context of their bachelor’s thesis under the supervision of the lead researcher. Importantly, they successfully passed the exams in psychodiagnostics before contributing to the data collection and were trained in the administration and scoring of the tests by qualified neuropsychologists.

**Table 1:** Descriptive statistics of the norm scores of Study 1.


	VCI	PRI	WMI	PSI	FS_IQ	NLV_IQ

Valid	30	30	30	30	30	30

Mean	101.967	105.567	104.833	93.467	102.133	93.600

Std. Deviation	11.312	10.621	15.412	16.126	12.153	7.356

Minimum	81	87	83	66	79	78

Maximum	124	127	143	122	123	114


Verbal Comprehension (VCI), Perceptual Reasoning (PRI), Working Memory (WMI) and Processing Speed (PSI), WAIS-IV Full-Scale IQ (FS_IQ), NLV-generated IQ-score estimate (NLV_IQ).

### Materials and procedures

Demographic information was recorded (age, gender, years of education) in a clinical interview. The NLV and WAIS-IV were then administered, in counterbalanced order according to standardized instructions described in the manuals of both tests. Data were collected from all participants in one session of about 2 hours, in sound attenuated testing rooms. The research complied with the guidelines of the Independent Ethics Committee of the Department of Applied Psychology of Thomas More.

#### Nederlandse Leestest voor Volwassenen (NLV; Schmand et al., 1992)

The Dutch version of the National Adult Reading Test (NART; Nelson, 1982) was used. This test consists of a list of 50 irregularly spelled words (organized from easy to difficult), which must be read out loud by the patient. Test sessions were recorded for relistening the pronunciation when needed and for a double check of the scoring by the other experimenter. Scoring was done according to the standardized scoring instructions (Ceuppens, 2014 Appendix B) and the norms of Mulder, Dekker & Dekker (1996) and were used to determine the NLV-generated IQ-score estimate. The internal consistency (Cronbach alpha) of the NLV within the current sample was 0.669 (after the removal of 14 items for which no variance between subjects was observed), indicating the NLV in the current study was measured with sufficient reliability (although this is lower compared to the alpha of 0.91 observed in the original study of Schmand et al. (Schmand et al., 1992)).

#### WAIS-IV-NL (Wechsler Adult Intelligence Scale- fourth edition, Dutch translation; Wechsler, 2012)

The Dutch version of the WAIS-IV was used to measure the intellectual functioning in healthy people. All 15 subtests were administered according to standardized instructions of the manual. The IQ- and index-scores were calculated according to the instructions described in the manual (meaning that only the first 10 subtests were considered). The Flemish normative data (Wechsler, 2012) were used to acquire the age-related subtest scores, the four index-scores (i.e., Verbal Comprehension, VCI; Perceptual Reasoning, PRI; Working Memory, WMI; and Processing Speed, PSI) and the total IQ.

### Analyses

To answer the question whether the NLV is still a useful tool to estimate premorbid intelligence, (Pearson) correlational analyses were conducted using JASP (2020) and bootstrap confidence intervals (using 1000 samples) around the correlation were calculated. The validity of the NLV was evaluated in two series of analyses. First, data obtained when using the norms were used to calculate the correlations between the NLV-generated IQ-score estimate and the WAIS-IV Full-Scale IQ and indices. Subsequently, to get an idea of the relation between these two tests independently from the norms, the latter analyses were repeated using the total raw score of the NLV (transformed to z-scores) and the raw scores on the WAIS-IV. To obtain a raw Full-Scale IQ estimate and raw index scores of the WAIS-IV, we first z-transformed the raw scores of all subtests. The raw Full-Scale IQ estimate was obtained by averaging the z-scores of all 10 relevant subtests that belong to this scale. The raw index scores were obtained by averaging the z-transformed raw scores of the relevant subtests that belong to a specific index. To give an impression of the data, the descriptive statistics (both processed and raw) were presented first. Subsequently, it was verified whether the variables were normally distributed, using the Shapiro-Wilks test. Finally, to answer the research questions, we reported the Pearson correlation coefficients (as all variables were normally distributed; *in both studies Shapiro-Wilks (30) > 0.933, all p’s > .063*) and associated Confidence Intervals (CI).

### Results

**Figure 1:**
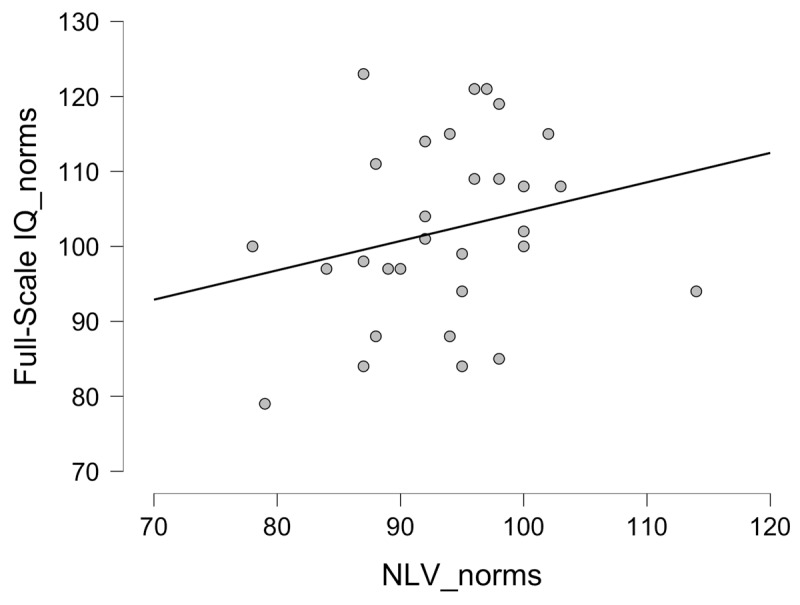
Scatterplot of the correlation between the NLV-generated IQ-score estimate (i.e., norm score) and the WAIS-IV Full-Scale IQ of Study 1. Note that the residuals (i.e., the distance of each data-point to the regression line) range from –18.857 to 23.452 (with a standard deviation of 11.807), indicating the imprecision of the NLV to estimate the WAIS-IV IQ.

The demographic information of Study 1 can be found in Table 1. The correlation between the NLV-generated IQ-score estimate and the WAIS-IV Full-Scale IQ was low *[r(30) = .237, p = .207; 95% CI = –.112–0.581; see Figure 1]*. This low correlation (and wide CI’s) clearly contrasts with similar correlations that were previously reported in the literature (typically ranging from .74 to .85, with comparable sample sizes; see Schmand et al., 1992).

More thorough analyses of the correlations between the various WAIS-IV indices and the NLV-generated IQ-score estimate did not reveal any significant relation either *[all the r’s < .299 and all p’s > .109]*. Table 2 provides an overview of the observed correlations and CI’s.

**Table 2:** The Pearson correlations of Study 1.


			N	PEARSON’S R	P	LOWER 95% CI	UPPER 95% CI

**Norms**							

NLV-IQ	–	VCI	30	.175	.355	–.167	.521

NLV-IQ	–	PRI	30	.097	.612	–.236	.450

NLV-IQ	–	WMI	30	.298	.110	–.069	.635

NLV-IQ	–	PSI	30	.136	.474	–.160	.467

NLV-IQ	–	FSIQ	30	.237	.207	–.112	.581

**Raw data (z-scores)**							

NLV	–	VCI	30	.132	.487	–.220	.455

NLV	–	PRI	30	.112	.556	–.238	.464

NLV	–	WMI	30	.341	.065	–.032	.620

NLV	–	PSI	30	.167	.377	–.113	.439

NLV	–	FSIQ	30	.248	.187	–.098	.563


*Note*: For all tests, the alternative hypothesis specifies that the correlation is positive, CI refers to the credible interval. NLV-generated IQ-score estimate (NLV-IQ), WAIS-IV Full-Scale IQ (FSIQ), Verbal Comprehension (VCI), Perceptual Reasoning (PRI), Working Memory (WMI) and Processing Speed (PSI).

Interestingly, the lack of correlations was not due to the used norms as the correlational analyses on the raw scores, mimicked those of the analyses on the data obtained when using the norms (see Table 2). Overall, the present findings thus indicate that in this age group, the performance on the NLV cannot be considered as a valid estimate of intelligence anymore.

### Discussion

The results of Study 1 indicate that the NLV has lost its power to predict the premorbid intellectual functioning in the age group of 20 to 29 years old, and that this is not caused by inappropriate norms: No evidence was found for the presence of a significant correlation between the NLV and the WAIS-IV IQ nor for the index scales, both when calculated on norm scores or on the raw data. It is important to note that the lack of correlations is unlikely to be caused by low reliability of the measurements. The Cronbach alpha (.669) of the NLV is within an acceptable range, and both tests were administered by trained experimenters. Furthermore, the lack of significant correlations is unlikely to be due to low between subject variability in intelligence. An inspection of Figure 1 learns that the average Full-Scale IQ of the WAIS-IV and standard deviation are within the range that could be expected from a representative sample. Although speculative, the high proportion of NLV-items for which no or little variance was measured could be an explanation for the lack of convincing correlations. In total 14 (out of 50) items were correctly or wrongly pronounced by all participants and in an additional 11 items only 1or 2 subjects made mistakes). Remarkably, this were not only the easier items. Some words that should be more difficult were also pronounced correctly by all subjects (see https://osf.io/qn5gv/ for raw and processed data; see Appendix A for an additional analysis of the discriminative power of the individual items). Although speculative, there could be several reasons why many of the NLV words have lost their discriminative power (changes in education, increased knowledge of other languages, the use of more foreign words (by young people), etc.). An important limitation of the current experiment is the age of the participants in this group (average age was 23.633 year (SD = 2.414; range 20–29 years). Remember that the NLV is composed in the late eighties/ early nineties of the previous century (Schmand et al., 1992) in a period before the participants of the current sample were born. Since language is dynamic over time (Crystal, 2012), it is possible that the current conclusions only apply to young people. For this reason, we repeated Study 1 in an older age group. We opted for the age range 45–65 because this is the age group for which the NLV is frequently used in the neuropsychological clinic (since the chance to acquired brain damage increases with age, and that (in Flanders) several rehabilitation centers preferentially treat patients below the age of 65). Furthermore, this age group was in-between 16 to 36 years old when the NLV was published (Schmand et al., 1992) and were thus young adults when the words of the test were selected.

## Study 2: The Validity of the NLV in 45–65 Year Old’s

### Participants

Fifty-one subjects participated in this study (30 females, 21 males). All participants had Belgian nationality and Dutch as native language. Their average age was 55.137 years (SD = 5.407; range 45–65 years). This sample was heterogeneous in terms of intelligence, as the average WAIS-IV Full-Scale IQ was 103.633 [SD = 12.950; range between 77 and 129; see Table 3]. The subjects self-declared that they had no history of neurological or psychological problems and had normal or corrected-to-normal vision. They all participated voluntarily without financial compensation and filled in an informed consent before participation. The participants were recruited and tested by four students in applied psychology of Thomas More and contributed to the study in the context of their bachelor’s thesis or internship. Importantly, they successfully passed the exams in psychodiagnostics before contributing to the data collection and were trained in the administration and scoring of the tests by qualified neuropsychologists.

**Table 3:** Descriptive statistics of the norm scores of Study 2.


	VCI	PRI	WMI	PSI	FS_IQ	NLV_IQ

Valid	51	49	51	51	49	51

Mean	104.392	101.429	99.765	105.843	103.633	103.353

Std. Deviation	12.025	14.487	14.133	13.584	12.950	10.709

Minimum	79	75	61	78	77	84

Maximum	131	135	129	138	129	124


Verbal Comprehension (VCI), Perceptual Reasoning (PRI), Working Memory (WMI) and Processing Speed (PSI), WAIS-IV Full-Scale IQ (FS_IQ), NLV-generated IQ-score estimate (NLV_IQ).

### Materials and procedures

The data were collected in the context of a larger study. For this reason, the NLV and WAIS-IV (see the materials section of Study 1 for a more elaborate description of both tasks) were administered together with other tasks (a revision of the Questionnaire intellectual status (Vragenlijst Intellectuele Status (VIS); Mas, 1979; and the Raven’s Progressive Matrices (RPM); Raven, 2006)). The data of these tasks were not considered in this study. A test session started with a clinical interview, where demographic information was collected (age, gender, years of education). Subsequently, the NLV, WAIS-IV, VIS and RPM were then administered according to the instructions described in the manuals (the order counterbalanced over participants). The internal consistency (Cronbach alpha) of the NLV within the current sample was 0.715 (after the removal of 10 items without interindividual variability), indicating the NLV in the current study was measured with sufficient reliability. All 15 subtests of the WAIS-IV were administered according to standardized instructions of the manual for 49 subjects. The index-scores were calculated according to the instructions described in the manual (meaning that only the first 10 subtests were used). The subtests of the PRI were omitted in 2 subjects, consequently, this index score and the Full-Scale IQ were not calculated for them. The research complied with the guidelines of the Independent Ethics Committee of the Department of Applied Psychology of Thomas More.

### Analyses and results

The same analyses were performed as in Study 1. The descriptive statistics of Study 2 can be found in Table 3.

**Figure 2:**
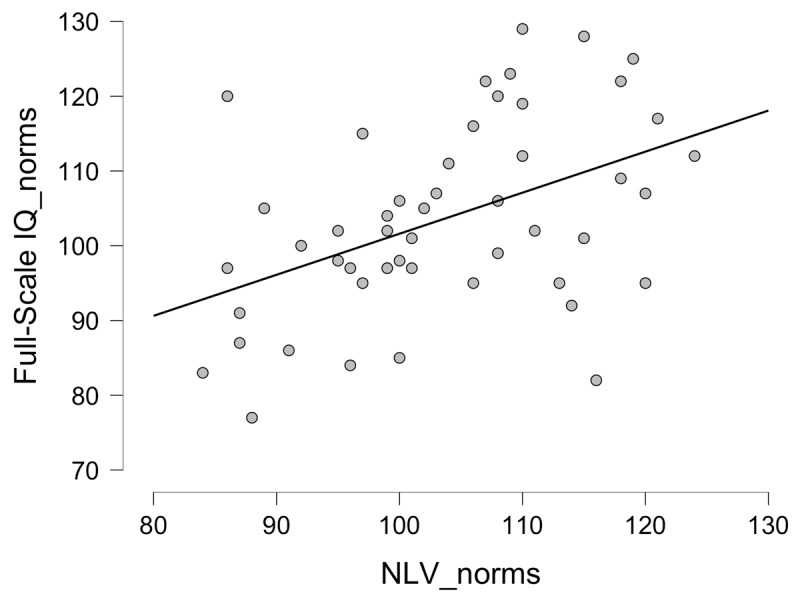
Scatterplot of the correlation between the NLV-generated IQ-score estimate (i.e., norm score) and the WAIS-IV Full-Scale IQ of Study 2. Note that the residuals (i.e., the distance of each data-point the regression line) range from -28.405 to 26.007 (with a standard deviation of 11.515), indicating the imprecision NLV to estimate the WAIS-IV IQ.

The Pearson correlation between the NLV-generated IQ-score estimate and the WAIS-IV Full-Scale IQ was *[r(49) = .457, p < .001; 95% CI = .175*–*.671; see Figure 2]*. More thorough analyses of the correlations between the different WAIS-IV Index-scores and the NLV-generated IQ-score estimate revealed significant correlations *[all r’s > .400, all p’s < .004]*, except for the correlation with the Perceptual Reasoning Index (PRI) for which the correlation was not significant *[r(49) = .207, p = .153]*. Interestingly, these correlations were slightly higher when using the raw data. Now, the correlation between the total raw score of the NLV and the total raw score of the WAIS-IV Full-Scale IQ (transformed to z-score) was *[r(49) = .549, p < .001; 95% CI = .263–.748]*. The correlations with the different index-scores revealed a similar pattern. All correlations except for the one of the PRI *[r(49) = .268, p = .062]* were significant *[all r’s >. 523; all p’s >.003]*. Table 4 provides an overview of the observed correlations in this study.

**Table 4:** The Pearson correlations of Study 2.


			N	PEARSON’S R	P	LOWER 95% CI	UPPER 95% CI

**Norms**							

NLV-IQ	–	VCI	51	.411	.003	.202	.607

NLV-IQ	–	PRI	49	.207	.153	–.134	.505

NLV-IQ	–	WMI	51	.404	.003	.156	.596

NLV-IQ	–	PSI	51	.433	.002	.167	.652

NLV-IQ	–	FSIQ	49	.457	<.001	.175	.671

**Raw data (Z-scores)**							

NLV	–	VCI	51	.523	<.001	.294	.704

NLV	–	PRI	49	.268	.062	–.054	.532

NLV	–	WMI	51	.426	.002	.211	.590

NLV	–	PSI	51	.444	.001	.152	.655

NLV	–	FSIQ	49	.549	<.001	.263	.748


*Note*: For all tests, the alternative hypothesis specifies that the correlation is positive, CI refers to the credible interval. NLV-generated IQ-score estimate (NLV-IQ), WAIS-IV Full-Scale IQ (FSIQ), Verbal Comprehension (VCI), Perceptual Reasoning (PRI), Working Memory (WMI) and Processing Speed (PSI).

### Discussion

After Study 1, which indicated that the NLV has lost its power to predict the premorbid intellectual functioning in the age group of 20 to 29 years old, we investigated the validity of the NLV in an older age group (between 45 and 65 years old). The results of Study 2 show that, although the observed correlations between the NLV-generated IQ-score estimate and the WAIS-IV Full-scale IQ and index scores, were higher *[r’s between .207 and .457]* compared to Study 1, they were still substantially lower compared to the correlations reported in the literature (typically ranging from r = .69 to r = .81; Bright et al., 2018; Lezak et al., 2012). Despite being larger (and in the expected direction), the correlations are well below the typically held psychometric standards used in test development of r < .70 (Pallant, 2010). Furthermore, a closer inspection of Figure 2 learns that some people with a low NLV-generated IQ-score estimate have a high WAIS-IV Full-Scale IQ and vice versa. This is particularly problematic for clinical practice. Furthermore, this indicates that the lower correlations are not the result of a systematic under or over estimation of the higher or lower IQ’s (Bouma et al., 2012). Analyses on the raw data revealed that the low correlations were also not due to the use of the norms of both tests. Although the correlations on the raw data were larger *[r = .549 vs. r = .457]* they were still not in the expected order of magnitude. In addition, the low correlations are unlikely be due to insufficient interindividual differences as, also this older sample was heterogeneous in terms of intelligence, as the average WAIS-IV Full-Scale IQ and standard deviation are with the range that could be expected from a representative sample. Finally, there are no indications to attribute the lower correlation to poor test reliability as the internal consistency of the NLV was sufficient (although there were again 16 words without (much) individual differences in their pronunciation; 10 without and 6 where only 1 or 2 mistakes were made; see Appendix A for an additional analysis of the discriminative power of the individual items), and the tests administered by trained experimenters who passed their exam in psychodiagnostics. So altogether, we conclude that also in this older age group the NLV has lost its power to properly predict someone’s premorbid level of intellectual functioning and cannot be used (anymore) to make a statement about a specific individual, as desired in practice when we go through a diagnostic process with a client.

## General Discussion

In the current study we re-evaluated the validity of the Dutch Reading Test for Adults (Nederlandse Leestest voor Volwassenen, NLV), a commonly used neuropsychological test to estimate premorbid intelligence (Schmand et al., 1991, 1992). Despite its widespread use in both clinical and scientific fields, validation studies and the collection of norms for the NLV date back to 1996 (Mulder et al., 1996) which is a long time as psychometric properties of tests can deteriorate over time (Evers et al., 2010).

Here, the validity of the NLV was evaluated in two samples without a neurological history who differed in age: 20–29 years (Study 1) and 45–65 years (Study 2). Whereas older studies with the NLV, and more recent studies with the NART (the English version of the task) showed high correlations in comparable experimental setups (in the range of .64 to .85; Bright et al., 2018; Lezak et al., 2012; Schmand et al., 1992) the results of the present studies indicate that the NLV has lost its validity in the Flemish population. The results in young healthy adults show that their scores on the NLV (both processed and raw) are not correlated to the measurements of the actual intelligence anymore (as measured with the WAIS-IV). For the older age-group, statistically significant correlations between both tests were found but were clearly below the .70, which is typically seen as a minimal correlation to ascertain sufficient validity (Pallant, 2010). Furthermore, correlations of around .50 (as observed here) only reflect around 25% of explained variance, making it unlikely that the NLV allows an accurate estimation of someone’s premorbid intelligence. Indeed, as can be seen in both Figures 1 and 2, the NLV often provides imprecise IQ estimations with over and under estimations ranging from 18 to 28 full-scale IQ points (with a SD of 11.500 IQ points). Importantly, the low correlations in both studies were due to imprecision over the entire range of the full-scale IQ: people with high scores on the WAIS-IV could obtain both high and low scores on the NLV, and vice versa. There is thus clearly a need for an updated and improved edition of the test. In other words, based on our findings, we advise against the use of the current NLV in clinical contexts (where this measurement is typically used for prognosis, setting up rehabilitation programs or for insurance related issues of an individual patient) or in neuropsychological research (where the NLV is often used as a co-variate to level-out differences in premorbid intelligence between different (patient) groups).

An open question is why the NLV has lost its validity? The NLV was originally developed and normed to be used in the Netherlands. As Dutch is the official language in Flanders too, the test is frequently used there. Although Flemish norms were (to our knowledge) never collected, Ceuppens (2014, Appendix B & C) updated the scoring procedures to fit better the Flemish pronunciation. Here, we scored the NLV according to this procedure, since all our subjects were living in Flanders. The low validity is thus unlikely due to differences between Dutch that is spoken in the Netherlands and Dutch that is spoken in Flanders. Another cause for the low validity is the inappropriate and outdated norms. This is however also not likely, as correlations with the raw scores of both tests are also too low to reach the necessary psychometric standards. This leaves the possibility that something might be wrong with the test itself. Although speculative, it is possible that words used in the NLV are outdated and have therefore lost their discriminatory power. Afterall our data has been collected in between 2014 and 2018, which is 20 to 26 years later than the publication of the NLV (which was in 1992). For example, in both Study 1 and 2, almost no interindividual variability was found in respectively 50% and 32% of the words (words that were pronounced correctly or incorrectly by almost everybody, see Appendix A). As a result, the task could have lost granularity to capture individual differences in a sensitive manner. This can however not be the entire story, as some individuals who score low on the NLV obtained high scores on the WAIS-IV, and vice versa. Some of the NLV items must thus have no or a negative correlation with intelligence. Future research will be needed for an in-depth investigation and the eventual update of (some of) the words used in the task. For this endeavor, factors like e.g., word frequency, age of acquisition, whether words are borrowed from other languages and/ or have many neighbors (and a combination of these factors) are worth considering. Luckily, nowadays several databases exist which contain up-to-date information for the Dutch language (see e.g., http://crr.ugent.be/programs-data/word-ratings). It is important to note that we don’t claim that the ability to read irregularly spelled words is no longer a good tool to estimate premorbid intelligence. Indeed, recent studies with the English version of the task still show that the principle still holds true today (Bright et al., 2018). Updating the NLV at the item level can thus be a promising endeavor and will be of great value for the clinical practice and the neuropsychological researchers who are now lacking a fast and easy tool to estimate premorbid functioning (see van der Linde & Bright, 2018, for an algorithm to find the optimal set of words).

Finally, we validated the NLV with the WAIS-IV, while Schmand et al. (1992) used the WAIS. In principle, the low correlations found in the current study could be attributed to the use of this newer version. Although there are substantial differences between the different versions of the WAIS (with differences at the level of the items, the subtests, and the index scores), direct comparisons between them, typically reveals high correlations between the corresponding indices. To our knowledge, the Dutch version of the WAIS-IV has never been directly compared to the WAIS itself, only to the WAIS-III (Pearson Assessment & Information BV, 2012). Of particular interest for the current study are the high correlations between the Total IQ (WAIS-III) and the full-scale IQ (WAIS-IV) *[r = .90]* and between the Verbal IQ (WAIS-III) and the Verbal Comprehension Index (WAIS-IV) *[r = .84]*. In other words, although the WAIS-III is of course not identical to the WAIS, we believe that it is unlikely that the low correlations found here are due to the differences between both versions of the WAIS (see also Bright et al. 2018, who did find sufficiently high correlations between the NART and the WAIS-IV). It is important to note that currently the WAIS is not used anymore in the clinical context, and that theoretically the concept of intelligence has much developed since the introduction of the WAIS. Thus, even in case the NLV would nowadays still correlate with the WAIS, the (practical) implications of this correlation would be limited.

In summary, the results of the current study show that the validity of the Dutch reading test for adults does not reach the required psychometric standards anymore to be used in clinical practice or scientific research. As such, we would advise against using the current version of the NLV to estimate premorbid performance level. Compiling a new word list will be necessary to make the concept behind the NLV operate again.

## Additional File

The additional file for this article can be found as follows:

10.5334/pb.1136.s1 Appendices. Appendix A to C.
