# Supplementary material for: The “Dutch Reading Test for Adults” has Been Used for 29 Years to Estimate the Premorbid Performance Level, does it Still Meet the Expectations?
Source: Psychol Belg. 2022 Aug 22;62(1):241–51. doi: 10.5334/pb.1136 (PMC9414804; doi:10.5334/pb.1136)
Supplement: Appendices. — Appendix A to C. [file pb-62-1-1136-s1.pdf]

APPENDIX A

Item analyses of the NLV for Study 1 (age range 20-29) and Study 2 (age range 45-65). The different Qs refer to the quartiles based on the Full-Scale IQ of the WAIS-IV (with Q1 reflecting group with the 25% lowest and Q4 the 25% highest IQ scores). The values in the table are the proportion of subject in each quartile group who pronounced that specific word correctly. For a word with good discriminatory power, this proportion, increases with increasing IQ.

	cadeau		sorry		junior		show		cito	
	Study 1	*Study 2*	*Study 1*	*Study 2*	*Study 1*	*Study 2*	*Study 1*	*Study 2*	*Study 1*	*Study 2*
Q1 (0-25%)	1,00	1,00	1,00	1,00	0,63	0,92	1,00	1,00	1,00	1,00
Q2 (25%-50%)	1,00	1,00	1,00	1,00	0,86	0,85	0,86	1,00	1,00	1,00
Q3 (50%-75%)	1,00	1,00	1,00	1,00	0,88	0,73	1,00	1,00	1,00	1,00
Q4 (75%-100%)	1,00	1,00	1,00	1,00	1,00	0,50	1,00	1,00	0,86	1,00

	shock		lyceum		fiasco		cacao		fair	
	Study 1	*Study 2*	*Study 1*	*Study 2*	*Study 1*	*Study 2*	*Study 1*	*Study 2*	*Study 1*	*Study 2*
Q1 (0-25%)	1,00	1,00	0,88	0,92	1,00	1,00	0,13	0,77	1,00	0,92
Q2 (25%-50%)	1,00	1,00	1,00	0,92	1,00	1,00	0,14	0,46	1,00	1,00
Q3 (50%-75%)	1,00	1,00	0,88	1,00	1,00	1,00	0,13	0,73	1,00	1,00
Q4 (75%-100%)	1,00	1,00	0,86	0,92	1,00	1,00	0,14	0,75	1,00	1,00

	ciruclaire		claim		daisy		cynicus		shuttle	
	Study 1	*Study 2*	*Study 1*	*Study 2*	*Study 1*	*Study 2*	*Study 1*	*Study 2*	*Study 1*	*Study 2*
Q1 (0-25%)	0,88	0,92	1,00	0,77	1,00	0,69	0,88	0,92	1,00	1,00
Q2 (25%-50%)	1,00	1,00	1,00	0,69	0,86	0,69	1,00	1,00	0,71	1,00
Q3 (50%-75%)	1,00	1,00	1,00	0,91	1,00	0,82	1,00	1,00	0,88	1,00
Q4 (75%-100%)	1,00	1,00	1,00	1,00	1,00	0,92	1,00	1,00	0,71	1,00

	fancy		cockney		punchbowl		adult		turquoise	
	Study 1	*Study 2*	*Study 1*	*Study 2*	*Study 1*	*Study 2*	*Study 1*	*Study 2*	*Study 1*	*Study 2*
Q1 (0-25%)	1,00	1,00	1,00	0,85	1,00	0,92	0,88	1,00	0,88	0,92
Q2 (25%-50%)	0,71	1,00	1,00	1,00	0,71	0,62	0,57	0,85	0,71	0,85
Q3 (50%-75%)	0,88	1,00	1,00	1,00	0,88	1,00	0,63	0,91	0,75	0,91
Q4 (75%-100%)	0,71	1,00	1,00	1,00	1,00	0,75	0,71	1,00	0,57	0,83

	cowboy		accessoire		ouvreuse		acacia		buggy	
	Study 1	*Study 2*	*Study 1*	*Study 2*	*Study 1*	*Study 2*	*Study 1*	*Study 2*	*Study 1*	*Study 2*
Q1 (0-25%)	0,50	0,92	0,75	0,69	0,88	0,92	0,63	0,92	1,00	0,62
Q2 (25%-50%)	0,71	0,77	0,57	0,62	0,71	0,92	0,57	1,00	1,00	0,46
Q3 (50%-75%)	0,88	0,91	0,75	0,73	0,88	0,91	1,00	0,91	1,00	0,46
Q4 (75%-100%)	0,86	1,00	0,71	0,92	1,00	1,00	0,57	1,00	1,00	0,42

	titulair		farce		alinea		eucalyptus		hydrolyse	
	Study 1	*Study 2*	*Study 1*	*Study 2*	*Study 1*	*Study 2*	*Study 1*	*Study 2*	*Study 1*	*Study 2*
Q1 (0-25%)	1,00	0,92	0,88	0,92	0,88	0,85	1,00	1,00	1,00	1,00
Q2 (25%-50%)	1,00	1,00	0,86	1,00	0,71	0,77	1,00	0,92	1,00	0,92
Q3 (50%-75%)	1,00	1,00	1,00	1,00	0,88	1,00	1,00	1,00	1,00	1,00
Q4 (75%-100%)	1,00	1,00	1,00	0,92	1,00	1,00	1,00	1,00	1,00	1,00

	enzym		funiculaire		ethyl		forsythia		illusoir	
	Study 1	*Study 2*	*Study 1*	*Study 2*	*Study 1*	*Study 2*	*Study 1*	*Study 2*	*Study 1*	*Study 2*
Q1 (0-25%)	0,75	0,85	1,00	0,62	1,00	1,00	0,13	0,62	1,00	1,00
Q2 (25%-50%)	1,00	1,00	0,86	0,92	1,00	1,00	0,29	0,39	0,86	0,85
Q3 (50%-75%)	0,88	0,91	0,88	1,00	1,00	1,00	0,50	0,73	1,00	1,00
Q4 (75%-100%)	1,00	1,00	1,00	1,00	1,00	1,00	0,29	0,67	1,00	0,92

	clique		clairvoyance		claque		cumuluswolk		epoque	
Q1 (0-25%)	*Study 1*	*Study 2*	*Study 1*	*Study 2*	*Study 1*	*Study 2*	*Study 1*	*Study 2*	*Study 1*	*Study 2*
Q2 (25%-50%)	0,88	0,77	0,63	0,92	1,00	0,85	0,38	1,00	1,00	0,85
Q3 (50%-75%)	0,86	0,85	0,29	1,00	0,86	0,85	0,86	1,00	1,00	1,00
Q4 (75%-100%)	1,00	0,73	0,63	0,82	1,00	1,00	0,75	1,00	1,00	1,00
	1,00	0,83	0,57	1,00	1,00	0,92	0,71	1,00	1,00	1,00

	queue		cloaca		auxiliair		farynx		guano	
Q1 (0-25%)	*Study 1*	*Study 2*	*Study 1*	*Study 2*	*Study 1*	*Study 2*	*Study 1*	*Study 2*	*Study 1*	*Study 2*
Q2 (25%-50%)	0,50	0,15	0,63	0,46	0,63	0,46	0,50	0,62	0,75	0,85
Q3 (50%-75%)	0,29	0,39	0,57	0,92	0,29	0,69	0,29	0,77	0,43	0,54
Q4 (75%-100%)	0,25	0,64	0,88	0,91	0,50	0,73	0,75	1,00	0,50	0,73
	0,14	0,67	0,71	0,83	0,71	0,83	0,43	0,92	0,71	0,75

	duce		gigolo		oecytype		vivace		caudillo	
	Study 1	*Study 2*	*Study 1*	*Study 2*	*Study 1*	*Study 2*	*Study 1*	*Study 2*	*Study 1*	*Study 2*
Q1 (0-25%)	0,13	0,08	1,00	0,77	0,00	0,08	0,13	0,39	0,75	0,46
Q2 (25%-50%)	0,14	0,46	0,71	0,69	0,00	0,08	0,29	0,77	0,43	0,46
Q3 (50%-75%)	0,00	0,64	0,63	0,82	0,00	0,09	0,75	0,64	0,50	0,64
Q4 (75%-100%)	0,00	0,25	0,71	0,67	0,00	0,42	0,43	0,83	0,71	0,92

APPENDIX B

Manual for scoring items of the “Nederlandse Leestest voor Volwassenen” – Flemish version

(Piet Ceuppens, 2014)

Veel voorkomende Vlaamse uitspraakfouten

Stimuluswoord	Stimuluswoord	Stimuluswoord
CADEAU	cadEAU	
SORRY	sOrry	
JUNIOR	zjUnior	djunior
SHOW	sjOw	
CITO	sjIto of sIto	kito
SHOCK	sjOck	sgok
LYCEUM	lySEum	
FIASCO	fiAsco	
CACAO	cacAW	cacajo, cacaOO, caca-o
FAIR	fAIr	
CIRCULAIRE	circulAIr of circulAIrE	
CLAIM	clAIm	
DAISY	dAIsy	deezie
CYNICUS	sYnicus	
SHUTTLE	sjUttel	
FANCY	fEnsy	fAncy
COCKNEY	cOcknie	
PUNCHBOWL	pUnsjbOwl	pUnsjbol (= met harde ‘O’, zonder lichte ‘w’)
ADULT	adUlt	Adult (Engels uitgesproken)
TURQUOISE	turkwAze	turkwaas
COWBOY	cOWboy	cobboj
ACCESSOIRE	axessOIre	Assesoir, Axessoir, Assesoir-e
OUVREUSE	oevrEUse	oevreus, oevrUs (van euhm)
ACACIA	acAzia	
BUGGY	bUggy (doffe ‘u’) of bUggy (minder doffe u). Mag zowel met ‘g’ (van Duitse ‘gut’) als met zachte ‘g’ (van ‘goed’).	

TITULAIR	tItulair	
FARCE	fArs of fArsE	
ALINEA	alInea	
EUCALYPTUS	eucalIptus	
HYDROLYSE	hIEdrolIEse	
ENZYM	enzIEm	
FUNICULAIRE	funiculAIr of funiculAIrE	
ETHYL	ettIl of Eethíel (met nadruk op 'ie', maar met 'ie' ipv 'i') wordt ook getolereerd.	Éttil, EEthiel (met nadruk op 'ee')
FORSYTHIA	forSITSia	forsiTia, forsiSSia
ILLUSOIR	illusOIr	
CLIQUE	klIEk	
CLAIRVOYANCE	clairvoyAnsu (met de 'a' als 'e' in Franse 'ensemble')	clairvoyanSe
CLAQUE	klAk	
CUMULUSWOLK	cUmmuluswolk	
EPOQUE	epOk	
QUEU	kEU	kjoe (Engels uitgesproken)
CLOACA	cloAAca	cloAca (met korte 'a'), cloacA
AUXILIAIR	auxili-air	auxilAIR
FARYNX	fArinks	
GUANO	guAno (met zachte 'g')	guano (met Duitse g van 'gut')
DUCE	dOEtsj of Doetsjé	deuce (Engels uitgesproken)
GIGOLO	djIgolo (zachte 'g') of gigolo (2x 'g' van Duitse 'gut')	gigolo (2x zachte g), djIgolo (met 'g' van Duitse 'gut')
OECOTYPE	Ecotiepe	OEkotiepe, EUcotiepe, OIkotiepe
VIVACE	vivAtjé	
CAUDILLO	caudIljo (met L) of caudIjo (zonder L)	caudiLLo (harde L)

Zie eventueel ook: http://www.vrt.be/taal/taaldatabank

APPENDIX C

Comparison between Dutch (abbreviated NLV; Schmand et al., 1992) and Flemish version (Piet Ceuppens 2014) of the "Nederlandse Leestest voor Volwassenen "

Black is the Flemish correct scoring, Dutch version is in red included

Common Flemish pronunciation errors

Stimulusword	Phonetically CORRECT pronunciation	Phonetically INCORRECT pronunciation
CADEAU	cadEAU	
SORRY	sOrry	
JUNIOR	~~zjUnior~~ jUnior	dzjunior
SHOW	sjOw Dutch manual says nothing	
CITO	~~sjIto or sIto~~ cito	kito
SHOCK	~~sjOck~~	~~sgok~~ schok
LYCEUM	lySEum	
FIASCO	fiAsco	
CACAO	~~cacAW~~ cacaO	~~cacajo, cacaOO, caca-o~~
FAIR	~~fAIr~~ Dutch manual says nothing	
CIRCULAIRE	~~circulAIr or~~ circulAIrE	
CLAIM	~~clAIm~~ Dutch manual says nothing	klem
DAISY	dAIsy	~~deezie~~ dizzy
CYNICUS	~~sYnicus~~ cYnicus	
SHUTTLE	~~sjUttel~~ shUttle	Schutle
FANCY	~~fEnsy~~ fAncy	fAncy
COCKNEY	~~cOcknie~~ cOckney	
PUNCHBOWL	~~pUnsjbOwl~~ pUnchbowl	~~pUnsjbol (= harsh 'O', without soft 'w')~~ punsbol
ADULT	adUlt	~~Adult~~ eddult (English pronunciation)
TURQUOISE	~~turkwAze~~ turqOIse	~~turkwaas~~
COWBOY	cOWboy	~~cobboj~~
ACCESSOIRE	~~axessOIre~~ accessOIre	~~Assesoir, Axessoir, Assesoir-e~~
OUVREUSE	~~oevrEUse~~ ouvrEUse	~~oevreus, oevrUs (of 'euhm')~~
ACACIA	~~acAzia~~ acAcia	Asasia
BUGGY	bUggy ~~(dull 'u') or bUggy (less dull u). Can be used with both 'g' (from German 'gut') and with soft 'g' (from Dutch 'goed').~~	Loud (Dutch) G

TITULAIR	~~tItulair~~ titulAIr	
FARCE	~~fArs or fArsE~~ fArce	
ALINEA	alInea	alinEa
EUCALYPTUS	eucalIptus	eusaliptus
HYDROLYSE	~~hIEdrolIEse~~ hydrolYse	
ENZYM	enzIEm	enzeem
FUNICULAIRE	funiculAIr or funiculAIrE	finiculère
ETHYL	~~ettIl or Eethíel (emphasizing ’ie’, but using ‘ie’ instead of ‘i’) is also tolerated.~~ ethYl	~~Éttil, EEthiel (emphasizing ‘ee’)~~ ettil
FORSYTHIA	forSITSia	~~forsiTia, forsiSSia~~
ILLUSOIR	illusOIr	
CLIQUE	~~klIEk~~ cLIque	klikuu
CLAIRVOYANCE	~~clairvoyAnsu (with the ‘a’ as ‘e’ in Franse ‘ensemble’)~~ clAIrvoyAnce	~~clairvoyanSe~~ klaarvojanse
CLAQUE	~~klAk~~ clAque	Klakuu
CUMULUSWOLK	cUmmuluswolk	
EPOQUE	~~epOk~~ epOque	epokuu
QUEU	~~kEU~~ qUEUe	~~kjoe (English pronunciation)~~
CLOACA	cloAAca	~~cloAca (with short ‘a’), cloacA~~kloasa
AUXILIAIR	~~auxili-air~~ AUxiliAIr	~~auxilAIR~~ auksilèr
FARYNX	fArinks	farniks
GUANO	guAno (with soft ‘g’)	guano (with German ‘g’ van ‘gut’) guujano
DUCE	~~dOEtsj or Doetsjé~~ dUce	~~deuce (English pronunciation)~~ dzjoes
GIGOLO	~~djIgolo (soft ‘g’) or gigolo (2x ‘g’ from Germans ‘gut’)~~ gIgolo	~~gigolo (2x soft ‘g’), djIgolo~~ (with German ‘g’ van ‘gut’) manual says DUTCH
OECOTYPE	~~Ecotiepe~~ oEcotype	~~OEkotiepe, EUcotiepe, OIkotiepe~~
VIVACE	~~vivAtjé~~ vivAce	vivas
CAUDILLO	~~caudIljo (with L) or caudIjo (without L)~~ caudILLo	caudiLLo (LOUD L)
